# Giant rhinolith in the left nasal cavity: a case report and literature review

**DOI:** 10.1093/jscr/rjaf016

**Published:** 2025-01-22

**Authors:** Maria R Alabdulaal, Hussain J Aljubran, Sultan H Alruwaili, Abdulrahman T Subaih, Hussain S Albahrani, Ali Almomen

**Affiliations:** College of Medicine, Arabian Gulf University, Manama 973, Bahrain; College of Medicine, Imam Abdulrahman Bin Faisal University, Dammam 43221, Saudi Arabia; Department of Otolaryngology Head and Neck Surgery, Eastern Health Cluster, Dammam 43221, Saudi Arabia; College of Medicine, Imam Abdulrahman Bin Faisal University, Dammam 43221, Saudi Arabia; College of Medicine, Imam Abdulrahman Bin Faisal University, Dammam 43221, Saudi Arabia; Department of Otolaryngology Head and Neck Surgery, King Fahad Specialist Hospital, Dammam 43221, Saudi Arabia

**Keywords:** calcified nasal mass, rhinolithiasis, endoscopic surgery, giant rhinolith

## Abstract

Giant rhinoliths are uncommon, mineralized concretions that usually develop around an intranasal foreign substance in the nasal canal. These lesions frequently cause respiratory problems, foul-smelling discharge, and nasal blockage. Clinical examination, endoscopy, and radiological imaging are used to make the diagnosis, and surgical removal is the only effective therapy. A giant rhinolith was discovered in a 42-year-old female patient with metastatic breast cancer came with nasal blockage. After 6 months, there were no complications or recurrences following the surgical removal of the calcified tumor. The significance of taking rhinoliths into account in individuals who have prolonged symptoms and unilateral nasal blockage is demonstrated by this case study. Consequently, early diagnosis and management are necessary to avoid potential complications and ensuring an excellent outcome.

## Introduction

Giant rhinoliths are uncommon calcareous concretions developed in the nasal cavity, frequently caused by the mineralization of an intranasal foreign material [1]. Rhinoliths typically develop in the lower nasal cavity and are more prevalent in females [2]. These abnormal masses result in breathing problems, foul-smelling discharge, and unilateral nasal blockage [3]. The diagnosis usually entails a complete history, physical examination, and nasal endoscopy; radiographic methods including computed tomography (CT) scan and conventional radiography are essential for localization [4, 5]. If left untreated, this condition can cause major consequences, such as erosion of the nasal septum and maxillary sinus wall, rhinosinusitis, and palatal perforation [6].

Surgical excision is usually the preferred method of treatment, although the approaches vary according to the rhinolith's size and location. For total excision, a lateral rhinotomy approach could be required in some circumstances [7]. Remarkably, some giant rhinoliths could have been odontogenic, perhaps developing from residual dental cysts [8]. Herein, the aim of this study is to present a case of a giant rhinolith to highlight the significance of early diagnosis and management of this condition, despite its rarity.

## Case presentation

A 42-year-old female, who is a known case of breast cancer (ductal carcinoma in situ) that metastasized to the brain, lung, liver, and bone 9 years ago, and currently on chemotherapy (Pertuzumab, Trastuzumab, and Docetaxel) after completing the radiation therapy. The patient presented to the otolaryngology clinic with a 2-month history of on/off right ear blockage progressive, right sided progressive decrease in hearing, and bilateral non-pulsatile tinnitus. It was also associated with on/off right nasal obstruction, epistaxis, and runny nose. However, the patient denies the presence of ear pain or discharge. Otolaryngological clinical examination showed bilateral mild congestion in the nasal cavities with hard bloody white mass lying under the floor of the inferior turbinate of the left nasal cavity. Also, the otoscope showed intact tympanic membrane with clear external auditory canal bilaterally. Meanwhile, audiometry exam revealed right-sided mild conductive hearing loss with normal left-sided hearing.

**Figure 1:**
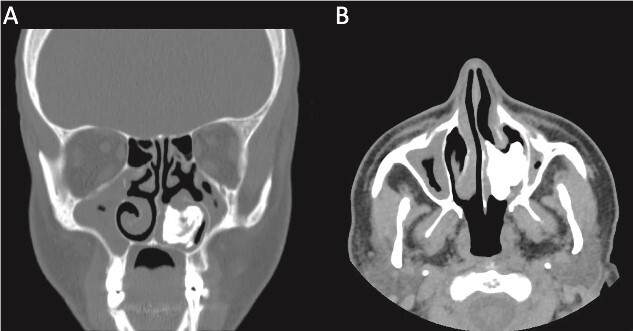
A CT scan with (A) coronal view and (B) axial view showing a well-defined expansile heterogeneous calcified/ossified lesion occupying the inferior aspect of the left nasal cavity.

**Figure 2:**
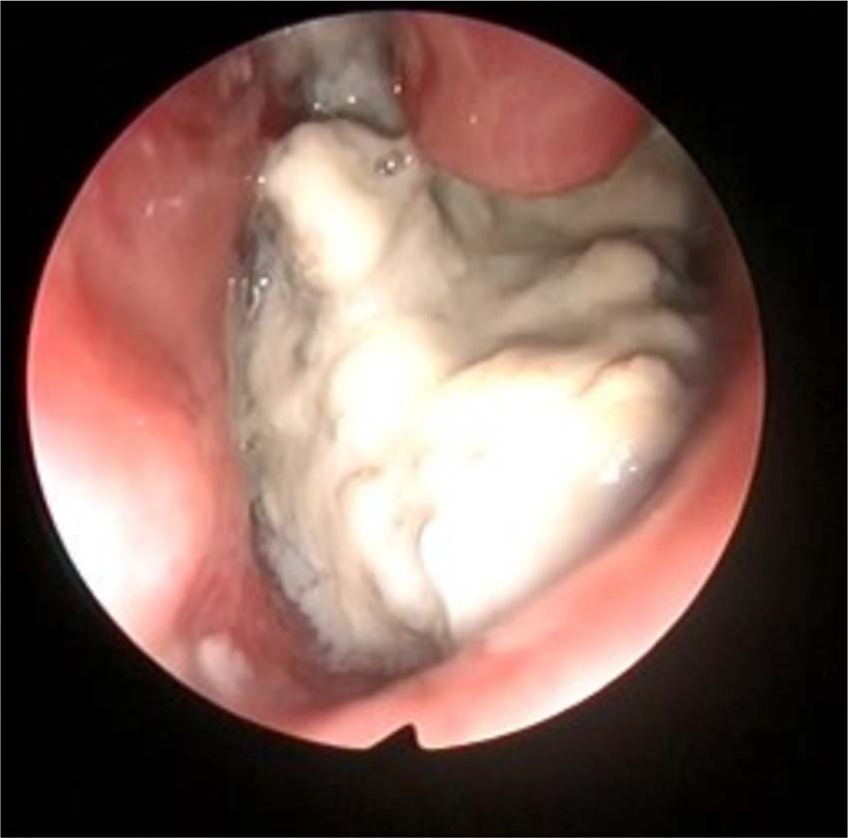
Intraoperative left nasal endoscopic view showing a whitish mass lying under the floor of the inferior turbinate.

**Figure 3:**
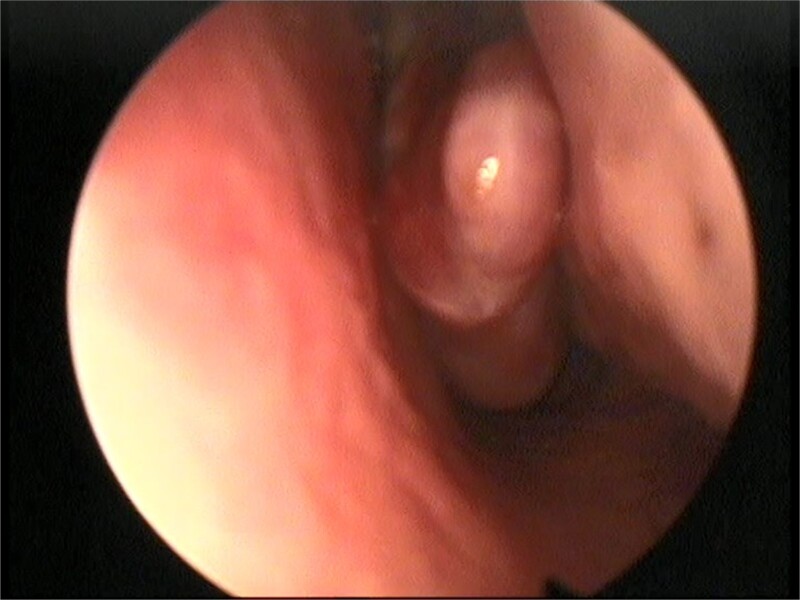
Postoperative nasal endoscopic view showing a clear left nasal cavity.

This presentation was clinically suggestive of left-sided rhinolith. Hence, a CT scan was obtained, which showed a well-defined expansile heterogeneous calcified/ossified lesion occupying the inferior aspect of the left nasal cavity measuring 2.5 × 3 × 2.1 cm (craniocaudal × anteroposterior × transverse). Also, the lesion was expanding the nasal cavities with bone remodeling of the medial wall of the maxillary sinus with remodeling and superior displacement of the left inferior turbinate (Fig. 1). These findings were highly suggestive of rhinolith; therefore, the patient was scheduled for a left endoscopic rhinolith removal. Intraoperatively, a giant, calcified nasal lesion was noticed and removed from the left nasal cavity, which was attached to the inferior and middle turbinates (Fig. 2). Furthermore, a biopsy from the lesion was taken for histopathological analysis, showing calcification with fibrin and hemorrhage. These findings confirmed the diagnosis of left nasal rhinolith, and the patient was discharged with no complications. Six months after the procedure, A the patient presented to the clinic with no signs of recurrence, and her endoscopic examination revealed clear nasal cavities (Fig. 3).

## Discussion

Giant rhinoliths are extremely uncommon, a number of factors are believed to be involved in the production of rhinoliths, despite the fact that the pathophysiology of these structures is still unknown [9]. The nucleus of these calculi might be external (like a bead) or endogenous (like blood or mucous) [2]. Rhinoliths are unilateral, often located on the nasal floor, and have a gray or brown appearance. They can be roughly spherical or irregular in form as described by Bansal *et al.* [10]. Furthermore, they can grow to considerable sizes.

A history of nasal blockage, discharge, odor, or discomfort is necessary for the diagnosis of rhinoliths. Additional signs and symptoms include headache, anosmia, epiphora, epistaxis, and facial or nasal edema [11]. Most often, rhinolith patients complain of unilateral nasal obstruction associated with foul-smelling, purulent, or blood-stained discharge from the same obstructed side; this is the typical presentation in such cases. Less often, they might present with swelling in the face or nose associated with anosmia, epiphora, or headache [12]. However, our patient presented with ear blockage, decrease in hearing, non-pulsatile tinnitus, nasal obstruction, epistaxis, and runny nose. Interestingly, the patient was a known to have a breast cancer; therefore, it was not a typical picture of rhinolith.

A CT scan is a crucial diagnostic tool for rhinoliths because of its sensitivity in identifying even minute amounts of calcification and its ability to determine the mass's size, shape, location, and extent as well as its relationship to the surrounding tissues. Depending on the type of nidus, they might appear radiographically as homogeneous or heterogeneous radiopacities with different sizes and shapes. They could have laminations, and occasionally their densities might be exceeding the surrounding bone [13]. The primary treatment for rhinolithiasis is surgery, which may be followed by medical intervention that involves nasal cavity disinfection with antibiotic and corticosteroid medication. Currently, the endonasal endoscopic approach is the preferred method [14].

## Conclusion

The presentation of a giant rhinolith is highlighted in this case study, underscoring the significance of prompt diagnosis and treatment in order to avoid its complications. Even though they are rare, rhinoliths should be taken into consideration when a patient presents with unilateral nasal obstruction or persistent nasal symptoms, particularly in areas with poor access to medical care. The rhinolith must be removed as soon as possible in order to relieve symptoms and avoid additional sinus or nasal damage.
